# Evaluation of the Cytotoxicity of Aqueous Extract and Oleo-Essential Oil of *Dorema ammoniacum* Plant Oleo-Gum Resin in Some Human Cancer Cell Lines

**DOI:** 10.1155/2022/9725244

**Published:** 2022-08-09

**Authors:** Pardis Mohammadi Pour, Spideh Bidad, Gholamreza Bahrami, Leila Hosseinzadeh, Mahdi Mojarrab, Mohammad Hosein Farzaei

**Affiliations:** ^1^Phytochemistry Research Center, Shahid Beheshti University of Medical Sciences, 981991953381 Tehran, Iran; ^2^Student Research Committee, Kermanshah University of Medical Sciences, Iran; ^3^Medical Biology Research Center, Medical School, Karmanshah University of Medical Sciences, Kermanshah 6714869914, Iran; ^4^School of Pharmacy, Kermanshah University of Medical Sciences, Kermanshah, Iran; ^5^Toxicology and Pharmacology Department, Faculty of Pharmacy, Kermanshah University of Medical Sciences, Kermanshah, Iran; ^6^Novel Drug Delivery Research Center, School of Pharmacy, Kermanshah University of Medical Sciences, Kermanshah, Iran; ^7^Medical Technology Research Center, Health Technology Institute, Kermanshah University of Medical Sciences, Kermanshah, Iran

## Abstract

**Results:**

Aqueous extract and essential oil reduced the viability of A549 cancer cells in a concentration-dependent manner. The lowest inhibitory concentrations (IC_50_) for both samples of *D. ammoniacum* oleo-gum resin were 10 and 2.5 *μ*g/ml for 24 hours in A549 cell line, respectively. After treatment with extract and essential oil of *D. ammoniacum* oleo-gum resin, ROS increased significantly compared to the control group. Although changes in caspase-3 did not show a significant increase in extract, the caspase-3 was found to be increased after exposure to essential oil and caspase-9 was downregulated after exposure to essential oil. Also, exposure to essential oil of *D. ammoniacum* caused a reduction in MMP level.

**Conclusion:**

Based on results, the cytotoxic effect of essential oil of *D. ammoniacum* can induce apoptosis toward A549 cell line via induction of oxidative stress, MMP depletion, and caspase-3 activation, which is independent to mitochondrial cytochrome c release and caspase-9 function.

## 1. Introduction

*Dorema* species is one of the member of the Apiaceae family, which belongs to the flora of Iran, among its seven species *D. ammoniacum* D. Don. and *D. aucheri* Boiss are only native to Iran [1]. This plant is one of the most important medicinal plants that is known in many arid and semiarid regions of Central Asia, at altitudes of 2500-900 meters in countries such as Iran, Afghanistan, and Pakistan [1]. The traditional usage of this herb includes decreasing the inflammation of the skin and is known to be useful as an analgesic, stimulant, laxative, chronic bronchitis, and persistent cough [2, 3]. In addition the antibacterial [4], antifungal [5, 6], and acetylcholinesterase [7, 8] inhibitory activities of *D. ammoniacum* resin have been reported [9], as well as its positive activity in treatment of melasma [10]. The essential oils and fruit oil which were obtained from *D. ammoniacum* demonstrated the strong cytotoxic activity on SW-480 and MCF-7 cells, human cancer cell lines, and high antimicrobial effects against *Bacillus subtilis* and *Staphylococcus epidermidis* [11–13].

Cancer is going to be a main cause of disease in the worldwide in the impending decades, and the incidence of the disease is anticipated to growth every year [14]. Cancer is the prior cause of mortality in developed countries and the second source of mortality in developing countries. More than 50% of cancer patents belong to developing countries, comprising those in Aisa and South America [15]. Cancerous cells demonstrate different strategies to restrict their death, and uncontrolled apoptosis is a vital mark in cancerous cell death [16]. The dysregulation in the function of mitochondria is one of apoptosis stimulant. Reactive oxygen species (ROS) generation generically regarded as apoptosis and cytotoxic inducers in cancerous cells [16]. Caspases are vital mediators of apoptosis. Through caspase protein, caspase-3 is a commonly activated death protease that catalyzed the specific cleavage of numerous critical cellular proteins. The pathways of activation of caspase-3 have been recognized as either independent of or dependent on caspase-9 function and mitochondrial cytochrome c release [17]. In this study, the essential oil and aqueous extract of *Dorema anmoniacum* were examined for their cytotoxic potential in human lung cancerous cell line (A549). The potency of cytotoxic extract against A549 cells was further evaluated for its ability to induce apoptosis, ROS generation, and MMP in A549 cells.

## 2. Material and Method

### 2.1. Plant Material, Extraction, and Essential Oil

*Dorema anmoniacum* oleo-gum resin was purchased from the medicinal plant market, and after its approval, a voucher number was PMP-823. With aim of preparing the oleo-gum resin extract (100 g), it was blended with water (1 L) by maceration method, and the next day after 24 hours of extraction, the aqueous phase was collected, water was merged once more, and then, the method was reiterated three times. The aqueous phase was filtered. In the final step, the water of the extract was entirely removed by rotary evaporator and was kept in -20°C in refrigerator for furthered investigation. In order to prepare the essential oil of *D. anmoniacum* oleo-gum resin, 50 g of oleo-gum resin with 200 mL of distilled water was placed in the Clevenger apparatus and the essential oil was prepared.

### 2.2. The Composition of the Essential Oil

The essential oil of oleo-gum resin was prepared by steam-distillation method via Clevenger apparatus. Then, it was analyzed using an Agilent 7890 GC (gas chromatography) of system equipped with a HP5 column (30 m × 0.25 mm, film thickness 0.25 *μ*m). The temperature column was increased computationally from 60°C to 250°C and maintained for 5 minutes at a rate of 3°C/min; it also was increased to 300°C and retained for 3 minutes at a rate of 10°C/min. The temperature of the injector was adjusted at 290°C. The carrier gas was Helium with a flow rate of 0.8 mL/min. Mass detector was Agilent 5973 with EI (electron ionization) system along with ionization energy of 70 eV. The ionization source temperature was adjusted to 220°C. For GC-MS analysis, hexane was used as a solvent to dissolve 1 *μ*L of the essential oil, and then, it was injected by the split mode (1 : 25). Electronic integration of the FID peak areas was applied for identification of the quantitative data. Agilent Technologies (MSD Chemstation software) was applied for data analysis. Components were identified by using their retention times (RT) to n-alkanes in comparison with those defined by Adams and Wiley 275 library [18, 19].

### 2.3. Cytotoxic Activity

#### 2.3.1. Cell Culture

A549 (human lung cancer), PC3 (human prostate cancer), HeLa (cervical cancer), and MCF7 (human breast cancer) were obtained from Pasteur Institute (Tehran, I.R. Iran). Cell lines were cultured in RPMI-1640 and supplemented with 10% fetal bovine serum, 100 *μ*g/ml of streptomycin, and 100 units/ml of penicillin and were remained at incubator at 37°C with 95% humidity and 5% CO2 [20].

#### 2.3.2. MTT Viability Assay

Cell viability was performed by applying the MTT assay (3-(4,5-dimethylthiazol-2-yl)-2,5-diphenyl tetrazolium bromide; Sigma). A549, PC3, HeLa, and MCF7 cells were seeded at 7 × 10^3^ cells in each well of a 96-well plate. After overnight incubation for cell attachment, the RPMI-1640 of each well was replaced with dissolve sample of extract in DMSO (20, 50, 100, and 150 *μ*g/mL) and dissolve sample of essential oil in DMSO (6.25, 12.5, 25, and 50 *μ*g/mL) and then incubated for 48 hours. Finally, 20 *μ*l of MTT (dissolved in PBS with concentration of 5 mg/mL) was added to per well and incubated at 37°C for 4 hours. Then, the medium was aspirated from the wells, and 200 *μ*l DMSO was admixed to each well for dissolving insoluble formazan. Finally, the absorbance values were measured by a Microplate Reader (BioTek Instruments) at wavelength of 490 nm to demonstrate the number of viable cells. The cell survival was determined according to the following equation [21]: cell survival = 100 × (absorbance of treated well–absorbance of blank)/(absorbance of control well–absorbance of blank). [22].

### 2.4. Measurement of Caspase Activity

To assess the caspase-3 activity, the cells were grown in 12 house plates, and then, the concentrations specified in IC_50_ for extract (10 and 20 *μ*g/mL) and for essential oil (1.5 and 3 *μ*g/mL) were added to the cells. Apoptosis was induced in cell lines. After 24 hours, the cells were separated from the plate using trypsin and transferred to the microtube. The microtubules containing the control cells and apoptotic cells were centrifuged at 4°C for 5 min, and the supernatant was aspirated. Then, 80 *μ*l of lysate buffer was added and placed in an ice bath for 30 minutes and then centrifuged at 4°C and 13000 rpm for 15 minutes, and the supernatant containing the proteins extracted from the cell was transferred to new microtube. After that, 80 *μ*l of assay buffer was added to each well along with 10 *μ*l of the substrate of each enzyme and 10 *μ*l of the enzyme. It should be noted that the protein content of the sample was calculated by the Bradford reagent [23].

The A549 cells were grown in 6 house plates, and then, the concentrations specified in IC_50_ for essential oil (1.5 and 3 *μ*g/mL) were added to the cells. Apoptosis was induced in cell lines. After 24 hours, the cells were separated from the plate using trypsin and transferred to the microtube. The microtubules containing the control cells were used to extract RNA by Parstous Total RNA Extraction Kit. To determine the caspase-9 expression in A549 cell line, the quantitative real-time RT-PCR was performed based on TaqMan methodology using the iCycler iQ™ Multicolor Real Time PCR Detection System (BIO-RAD, Hanover, MD, USA). The prepared primer is shown in Table 1. The following iCycler iQ run protocol was carried out for 10 min at 94°C, then for 25~35 cycles of 15 s at 95°C, 30 s at 50~60°C, 30~60 s at 72°C, and finally for 1 cycle 5 min at 72°C. The essential oil samples at the concentration of 1.5 and 3 *μ*g/ml were amplified in triplicate in a one-assay run. After the amplification, each sample had a Ct value which is the cycle number at which the fluorescence signal crosses the threshold. To calculate the relative fold gene expression of caspase-9, 2^-(∆∆Ct)^ was used for quantitative real-time polymerase chain reaction data analysis.

### 2.5. Measurement of Mitochondrial Membrane Potential

The mitochondrial membrane potential (MMP, *ΔΨ*m) was estimated by using a lipophilic cationic Rhodamine 123 (Rh123) probe [24]. Rh123 dye could penetrate the mitochondria selectively and constitute monomers that emit green fluorescence in which case MMP is relatively low or aggregates and emits red fluorescence in which case MMP is relatively high [25]. To measure *ΔΨ*m, cells implanted in a plate with 12 cells and then IC_50_ for extract (10 and 20 *μ*g/mL) and for essential oil (1.5 and 3 *μ*g/mL) and DMSO as controls were added. At the next step, they were incubated for 24 hours. Stock solution of Rh123 dye was prepared in DMSO. Rh123 with a concentration of 4 mM was prepared, and 15 ml was added to each well and kept in an incubator for half an hour. Then, the cell culture medium was removed, the cells were washed with cold PBS, and then, 1 mL of Triton-x100 was added to each well to lysis the cells and kept at 4°C for half an hour. In the next step, after centrifuging the cells at 13000 rpm, the amount of fluorescence of the cells was measured using a microplate reader equipped with fluorescence at an excitation wavelength of 488 nm and an emission wavelength of 520 nm. To measure the amount of protein, 3 *μ*L of the supernatant was added to 100 *μ*L of Bradford reagent and its absorbance was read at 630 nm. The amount of protein absorption of each sample should be placed in the standard protein equation (*Y* = 0.188 *X* + 0.31). Then, the amount of protein in each sample and the amount of absorption based on 1 mg of protein were calculated [25].

### 2.6. ROS Accumulation Assay

2′,7′-Dichlorofluorescin diacetate (DCFDA), a fluorogenic dye, was used to evaluate ROS levels. To measure ROS, after the cells in the main flask had reached a certain number, the cells were implanted in a 12-well plate. After treating with IC_50_ for extract (10 and 20 *μ*g/mL) and for essential oil (1.5 and 3 *μ*g/mL) and DMSO as controls, DCF was added to the cell culture medium and kept in the incubator for 30 to 45 minutes and shooked for 10 min. The contents of the wells were transferred to the microtube and centrifuged at 13000 rpm for 15 min. The amount of fluorescence of the cells was measured using a microplate reader equipped with fluorescence at an excitation wavelength of 480 nm and an emission wavelength of 528 nm. The plate used to measure ROS should be dark [26].

### 2.7. Statistical Analyses

Cell viabilities and IC_50_ values were expressed as mean ± standard deviation [27] of the mean. Statistical analysis was done using Tukey-Kramer test using the SPSS software version 20. The results were considered as significant when *P* < 0.05.

## 3. Results

In this study, after preparing the aqueous extract of *D. ammoniacum* oleo-gum resin, its dry powder was prepared and the yield of the dried extract compared to the primary dry weight of the plant was calculated to be 6.828% and for essential oil compared to the primary dry weight of the plant was 0.25%.

### 3.1. The Essential Oil Yield and Composition

The yield of isolation of the essential oil was 0.25%. Forty-two compounds were identified by GS-Mass analysis that demonstrated the 60.05% of the total essential oil. The chemical composition of this essential is depicted in Table 2. The main constituents of the essential oil were Cuperene (14.31%), *β*-Funebrene (12.74%), and Barbatene (9.21%).

### 3.2. Evaluation of Cytotoxic Activity

IC_50_ is the minimum concentration of the drug that inhibits the growth of 50% of the cell population. In order to obtain IC_50_, the effect of different concentrations of aqueous extract and essential oil of *D. ammoniacum* oleo-gum resin on the survival of A549 cells was investigated. Finally, cell survival was assessed using MTT assay.

**Figure 1:**
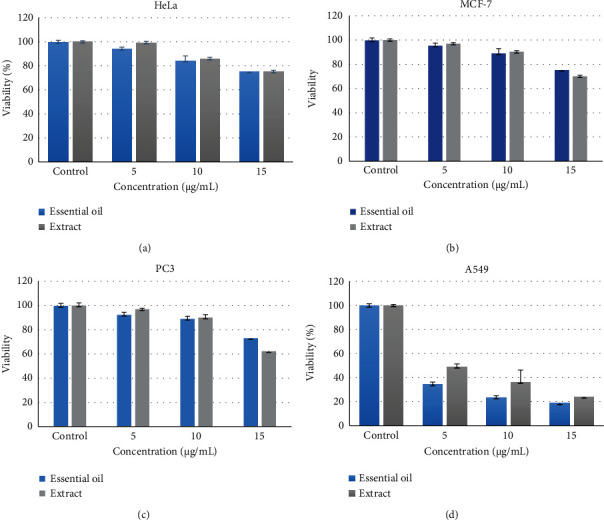
Cytotoxicity of *D. ammoniacum* oleo-gum resin extract and essential oil against cell lines. (a) HeLa cell line, (b) MCF7 cell line, and (c) PC3 cell line. (d) The effect of aqueous extract of *D. ammoniacum* oleo-gum resin on the survival of A549 cells. Survival was measured by MTT method. The results are expressed as mean ± SD of three independent experiments (*n* = 3).

According to the results of cytotoxicity (Figure 1), it was found that the IC_50_ to inhibit the growth of A549 cancer cells in the essential oil was at a concentration of 1.5 mg/mL and for the extract was at a concentration of 10 *μ*g/mL. Therefore, for the next steps, concentrations of 20 and 10 *μ*g/mL for the extract and concentrations of 3 and 1.5 *μ*g/ml for the essential oil were selected as the main concentration. Although higher concentrations could significantly inhibit the growth of cancer cells, but due to their high cytotoxic effects, concentrations of 10 and 1.5 *μ*g/mL for the extract and essential oil were defined as the best concentrations, respectively. Therefore, in order to investigate the apoptotic role of essential oil and ash extract, the mentioned concentrations were used in other experiments.

### 3.3. Evaluation of Caspase-3 and Caspase-9 Activity

**Figure 2:**
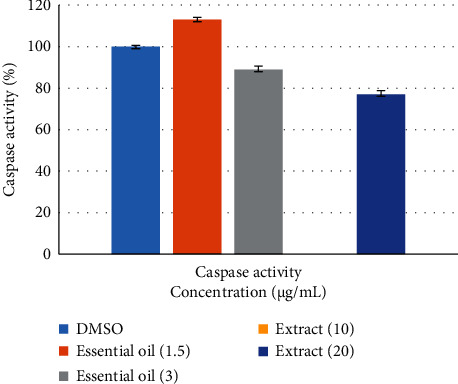
Effect of different concentrations of aqueous extract and essential oil of *D. ammoniacum* oleo-gum resin on caspase-3 activity in A549 cells. Caspase-3 activity was measured based on the p-nitroaniline colorimetric reaction, and the data were expressed as a percentage. The results are expressed as mean ± SD of three separate experiments (*n* = 3). Tukey-Kramer test was used to examine the statistical difference (^∗^*p* < 0.05 and ^∗∗∗^*p* < 0.001).

Activation of the caspase chain reaction is required to initiate apoptosis. One member of the caspase family is caspase-3, which is known to be a key mediator in the mitochondrial (internal) and death receptor (external) pathways of apoptosis [17]. Therefore, in this study, to show the occurrence of apoptosis, the activity of caspase-3 was investigated. According to Figure 2, in spite of the fact that the essential oil with a concentration of 1.5 *μ*g/mL has the ability to increase the activity of caspase-3 in A549 cell line, the extract was unable to increase the activity of this protein. In stark contrast, essential oil at IC_50_ concentration was able to increase caspase-3 activity compared to controls.

**Figure 3:**
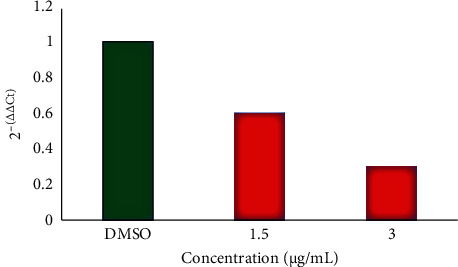
Effect of different concentrations of essential oil of *D. ammoniacum* oleo-gum resin on caspase-9 activity in A549 cells. Caspase-9 activity was measured based on the RT-PCR reaction, and the data were expressed as relative fold gene expression. The results are expressed as 2^-(∆∆Ct)^ of three separate experiments (*n* = 3).

One of the member of caspase family is caspase-9 that belongs to cysteine proteases that have been involved in cytokine and apoptosis processing. During the time that cells receive apoptotic signals, cytochrome c is released by mitochondria. In the next step, cytochrome c attached to Apaf-1, the mammalian Ced-4 homologue, together with dATP. The consequent complex leads to caspase-9 activation. Activation of caspase-9 protein results in cleavage of downstream caspases comprising caspase-3, caspase-6, and caspase-7 initiating the caspase cascade [28]. Therefore, in this study, to show the occurrence of apoptosis, the activity of caspase-9 was studied. According to Figure 3, the essential oil with a concentration of 1.5 and 3 *μ*g/mL both downregulated the caspase-9 expression in A549 cell line.

### 3.4. Electrical Potential Difference (*ΔΨ*m) across the Mitochondrial Membrane

**Figure 4:**
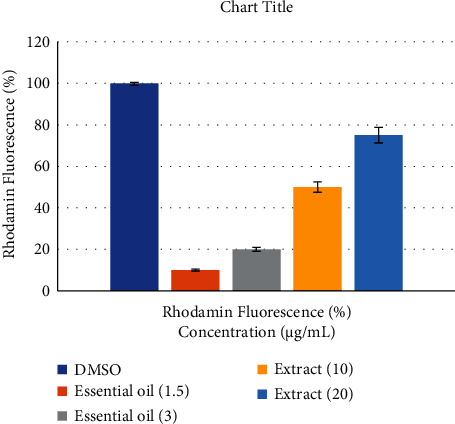
Effect of aqueous extract and essential oil of *D. ammoniacum* oleo-gum resin on MMP in A549 cells detected with Rh123 via fluorescence microplate reader. The results are expressed as mean ± SD of three independent experiments (*n* = 3). Turkey-Kramer test was used to examine the statistical difference.

Mitochondria play an important role in inducing apoptosis, so measuring the potential of mitochondrial membranes is very important in assessing apoptosis. Intracellular fluorescence indicates the amount of potential changes in the mitochondrial membrane. The results of this study showed that at a concentration of 3 *μ*g/mL of essential oil, a remarkable reduction in the potential of mitochondrial membrane was observed in A549 cell in comparison with the control group. On the other hand, aqueous extract of *D. ammoniacum* oleo-gum resin reduced the MMP at the concentration of 10 *μ*g/mL (Figure 4).

### 3.5. ROS Overproduction in A549 Cell

**Figure 5:**
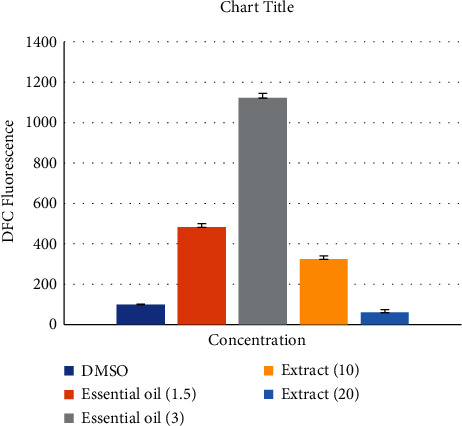
Effect of essential oil and aqueous extract of *D. ammoniacum* oleo-gum resin on the induction of ROS in A549 cells. The bar chart is the average fluorescence DCF. The results are expressed as mean ± SD of three separate experiments. ^∗^*p* < .05 and ^∗∗^*p* < .01 were considered significant, statistically.

**Figure 6:**
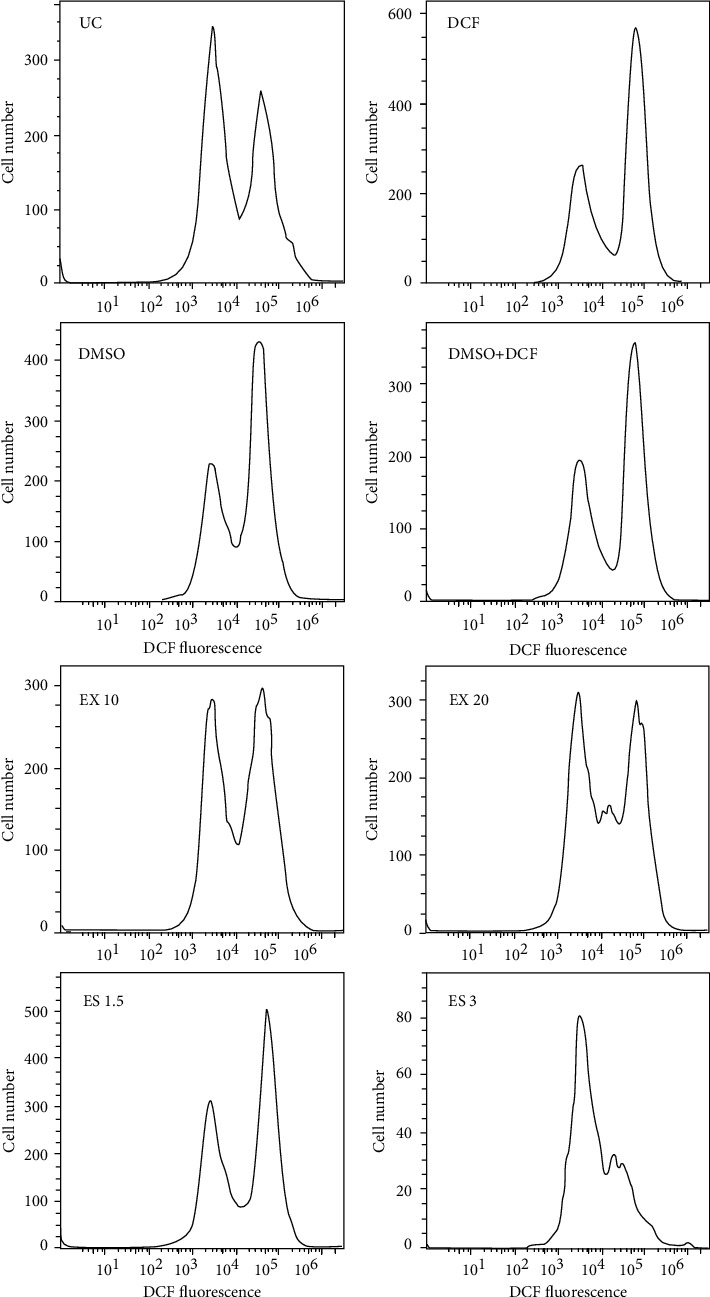
Histogram of ROS measured by flow cytometry (quantitative histogram due to changes in the mean fluorescent intensity of DCFH-DA probes compared to cells without extract and essential oil) (UC: untreated cell; DCF: cell with DCF; ES: essential oil; EX: extract).

Dichlorodifluorostat (DCDFA) reagent was used to determine ROS. After treating the cells with this reagent, it is hydrolyzed by esterase and converted to dichlorofluorescein, which can be oxidized by radical compounds to the fluorescent compound DCF [26]. As a result, the amount of fluorescence inside the cell indicates the ROS-mediate cytotoxicity inside the cell. Based on Figure 5, addition of aqueous extract and essential oil to the cells led to a significant increase in the amount of ROS in treated cells compared to the control group, and the amount of ROS in the essential oil showed the most increase at concentration of 3 *μ*g/mL (Figure 5). The histogram of ROS measured by flow cytometry is illustrated in Figure 6.

## 4. Discussion

Lung cancer death amid never-smoking men and women in CPS-II was 17.1 and 14.7 per 100,000 person-years [29]. Despite the discovery of numerous drugs and the remarkable advances in the treatment of cancer disease, many common treatments of these patients have serious side effects including toxicity and drug resistance [30]. Today, newer perspectives such as the induction of apoptosis in the treatment of this disease are considered [31]. Studies over the past decade have shown that almost a large number of drugs used to treat cancer induce the process of apoptosis in cancer cells, and apoptosis is the most important route of cell death, especially in solid tumors [31]. The present study was designed to evaluate the cytotoxicity and apoptosis caused by aqueous extract and essential oil of *D. ammoniacum* oleo-gum resin on A549 lung cancer cell line.

In the previous study that was conducted by Azade Raeesdana et al. in 2014, the acute toxicity of *D. ammoniacum* oleo-gum-resin solution (1250, 2500, and 5000 mg/kg) was studied on Wistar rats. The findings displayed no mortality, and the LD50 (Median Lethal Dose) was more than 5000 mg/kg. In addition, in subacute treatment, no significant changes were observed in hematological and biochemical parameters at any doses in comparison with the control group. Varying effects were observed in histopathological analysis of the organs. Histopathological analysis of the liver showed mild inflammation and vacuolar degeneration at 200 and 500 mg/kg doses. Histopathological analysis of the kidney displayed congestion of glomeruli and a widening of the urinary space at 500 mg/kg in comparison with the control group. It was concluded that the acute consumption of the oleo-gum resin of *D. ammoniacum* is not accompanied by the sign and symptoms of toxicity, while its consumption in long term could be associated with hepatotoxicity and renal toxicity [19]. In another study conducted by Yousefzadi et al. in 2011, the toxicity effect of *D. ammoniacum* essential oil was investigated on two cancer cell lines (MCF and SW480) and two normal cell lines (HFSF and HFLP). The results showed that essential oil had low toxicity, and both cancer cells were more sensitive than normal cells [12]. In 2019, Tavakoli and colleagues studied the embryonic vascular toxicity of *D. ammoniacum* on a membrane model of chicken embryos. Based on results, changes in vascular parameters and gene expression due to consumption of *D. ammoniacum* can eventually lead to fetal abnormalities. Therefore, consumption of this plant during the embryonic development period in doses higher than 50 mg/kg should be limited [32]. In a study conducted in 2014 by Morteza Eskandani and colleagues, they examined the apoptosis of phenolic compounds extracted from the *Dorema glabrum* Fisch. C.A. The results showed that diglucosyl caffeoyl ester has the cytotoxic effect and induced apoptosis in CAOV-4 cell line.

Oxidative stress is one of the factors that plays a significant role in inducing apoptosis in different cells [30]. Therefore, to investigate whether the increase in oxidative stress by the extract and essential oil of *D. ammoniacum* oleo-gum resin can induce apoptosis in lung cancer cells, a number of parameters involved in apoptosis such as caspase activity and MMP were investigated. In the present study, the parameters involved in apoptosis on A549 cell line showed that the essential oil of oleo-gum resin was able to induce apoptosis in lung cancer cells compared to the aqueous extract. *D. ammoniacum* oleo-gum resin extract induced apoptosis by reducing the potential of MMP, increasing oxidative stress, and increasing the activity of caspase-3 through the caspase-independent pathway and downregulating caspase-9 in A549 cell line. So it showed that the apoptosis did not happen via mitochondrion-mediated procaspase-activation pathway of caspase-9, and it happened through the death signal-induced and death receptor-mediated pathway [33]. Finally, according to the use of up-to-date and extensive techniques in the present study, it can be concluded that *D. ammoniacum* oleo-gum resin essential oil, due to its low toxicity, has the ability to induce apoptosis via caspase-3 activation, which is independent to mitochondrial cytochrome c release and caspase-9 function [34] and could be an effective supplementary in preventing or controlling lung cancer.

## 5. Conclusion

The findings of the present study showed that the essential oil and aqueous extract of *Dorema ammoniacum* oleo-gum resin led to cell death via apoptosis. ROS overproduction and activation of caspase-3, which is independent of mitochondrial cytochrome c release and caspase-9 function after treatment with *D. ammoniacum* oleo-gum resin essential oil, possibly is one of the triggers for apoptosis. On the other hand, decreasing the MMP in A549 cell lines is the other reason of the triggers of apoptosis. Moreover, further studies were required to address the impact of *D. ammoniacum* oleo-gum resin extract and essential oil against other human lung-cancer cell lines as well as its role in other underlying mechanisms. In addition, further studies using *in vivo* experimental models were also warrant.

## Figures and Tables

**Table 1:** Primers and probes of caspase-9.

Oligo name	Seq. (5-3)	Molecular weight	OD^1^ (1000 *μ*M)	Nmol^2^	TM^3^	GC%^4^
CAs3forward	GTTTGAGGACCTTCGACCAGCT	6726.4	2.3	11.28	62.12	54.55
CAs3reverse	CAACGTACCAGGAGCCACTCTT	6664.3	4.4	21.79	62.12	54.55

^1^Oligonucleotide absorbance at 260 nm. ^2^Amount of nucleotide based on nM. ^3^Temperature of melting point of nucleotide. ^4^Percentage of GC.

**Table 2:** The essential oil composition of *Dorema ammoniacum* of oleo-gum resin.

	Retention time	Area% A1	Compound
1	17.87	14.31	Cuparene
2	15,65	12.74	*β*-Funebrene
3	16,38	9.21	Barbatene
4	17,27	7.46	Germacrene D
5	10,98	6.68	Z-Ocimenone
6	17.92	4.91	Bisabolene
7	16,58	4.25	*α*-Humulene
8	16,67	3.85	Amorpha-4,11-diene
9	11.17	3.31	E-Ocimenone
10	8.34	3.23	2,6-Dimethyl3,5,7-octatriene-2-ol,Z,Z
11	17.18	3.13	*β*-Chamigrene
12	4.2	2.89	*α*-Pinene
13	16.33	2.5	Guaiadiene
14	11.02	2.43	Thymol methyl ether
15	15.03	2.38	*β*-Elemene
16	15.74	2.13	Trans-caryophyllene
17	17.71	1.83	Himachalene
18	18.24	1.68	*δ*-Cadinene
19	10.36	1.49	2,6-Dimethyl3,5,7-octatriene-2-ol,E,E
20	14.62	1.36	*α*-Copaene
21	16.45	1.31	*β*-Selinene
22	17.59	1.27	*α*-Selinene
23	16.73	0.56	Acoradiene
24	15.79	0.56	Cedrene
25	18.47	0.55	*γ*-Cuprenene
26	16.19	0.54	Elemene
27	4.96	0.45	*β*-Pinene
28	18.39	0.44	Dauca-4(11),8-diene
29	19.04	0.35	Dauca-4(11),8-diene
30	14.85	0.34	*β*-Burbonene
31	16.88	0.33	Aromadendrene
32	21.8	0.28	Bulnesol
33	17.38	0.27	*β*-Selinene
34	16.03	0.27	Thujopsene
35	8.1	0.24	2,6-Dimethyl1,3(E)-5(Z),octatetraene
36	19.9	0.14	Caryophyllene oxide
37	9.26	0.13	Dimercaprol
38	10.12	0.07	Myrtenol
39	13,92	0.05	*α*-Cubebene
40	5.88	0.04	p-Cymene
41	5.96	0.02	Limonene
42	4.06	0.01	*α*-Thujene

Total identified: 60.05.

## Data Availability

Data are available on request.

## Abbreviations

ROS: Reactive oxygen species
MMP: Mitochondrial membrane potential
GC: Gas chromatography
EI: Electron ionization
FID: Flame ionization detector
MS: Mass
RT: Retention times
MTT: (3-(4,5-Dimethylthiazol-2-yl)-2,5-diphenyl tetrazolium bromide
DMSO: Dimethyl sulfoxide
Rh123: Rhodamine 123
DCFDA: 2′,7′-Dichlorofluorescin diacetate.
